# Freshwater Discharge and Salinity Drive Taxonomic and Functional Turnover of Microbial Communities in a Turbid Macrotidal Estuary

**DOI:** 10.1111/1758-2229.70135

**Published:** 2025-07-14

**Authors:** Luz Amadei Martínez, Koen Sabbe, Sofie D'hondt, Renaat Dasseville, Ilse Daveloose, Tine Verstraete, Peter Chaerle, Natacha Brion, Tom Maris, Patrick Meire, Wim Vyverman

**Affiliations:** ^1^ Laboratory of Protistology and Aquatic Ecology, Department of Biology Ghent University Ghent Belgium; ^2^ Department of Archeology, Environmental Change and Geochemistry Vrije Universiteit Brussel Brussels Belgium; ^3^ ECOSPHERE Research Group University of Antwerp Wilrijk Belgium

**Keywords:** 16S, 18S, bacteria, metabarcoding, monitoring, protist

## Abstract

The drivers of spatiotemporal changes in microorganism's functional community structure remain poorly understood. Using DNA‐amplicon sequencing we studied the spatiotemporal dynamics of bacterial and eukaryotic microbial communities in the freshwater and brackish tidal reaches of the Schelde estuary (Belgium) from 2018 to 2021. Our analyses revealed pronounced seasonal and longitudinal turnover in autotrophic and heterotrophic microbiota, mainly driven by changes in freshwater discharge, which modulate the salinity and turbidity gradient. Higher discharge in early spring led to a more uniform community composition across the estuary, with higher relative abundances of heterotrophic eukaryotes. As discharge decreased in late spring, the salinity gradient and associated turnover in community composition became more accentuated, with Actinomycetota and diatoms dominating the upstream reaches, and ciliates, fungi and marine bacteria being relatively more important downstream from the maximum turbidity zone (MTZ). This study revealed fine‐scale turnover in (semi)cryptic phytoplankton taxa and spatiotemporal changes in parasitism linked to bloom termination. High discharge due to exceptionally heavy rainfall resulted in the disruption of the phytoplankton bloom, more downstream spreading of freshwater species and a decline in brackish and polyhaline species. These results emphasise the intricate link between hydrodynamics and microbial community dynamics and ecological functions in estuarine ecosystems.

## Graphical abstract

Seasonal shifts in microbial community composition in the Schelde estuary are controlled by the interaction between freshwater discharge and marine intrusion. However, these recurrent annual patterns can be disrupted by extreme precipitation events. This study highlights the complex link between hydrodynamics and microbial dynamics and ecological functions in estuarine ecosystems.

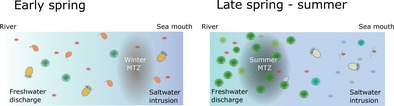

## 1 Introduction

Estuaries are among the most productive ecosystems on Earth (Winder and Cloern 2010), where the interaction between river discharge and seawater intrusion leads to strong physical (e.g., salinity, turbidity and temperature), chemical (e.g., inorganic and organic nutrients) and biological gradients (Jeffries et al. 2016; Cloern et al. 2017). At the convergence of longitudinal transport forces (freshwater discharge, marine intrusion and tidal pumping), flocculation is promoted and marine and fluvial sediments accumulate, forming areas of heightened turbidity known as maximum turbidity zones (MTZ) (Yu et al. 2014).

The MTZ is a pivotal feature in the ecological functioning of an estuary. It is an area where upstream organic matter and microorganisms accumulate (Lancelot and Muylaert 2011). In addition, because of high bacterial activity and the strong limitation of photosynthesis due to low light availability, this zone is frequently dominated by heterotrophs (Goosen et al. 1999). Heterotrophic microbes play a key role in the estuarine biogeochemical cycles through respiration and sequestration of fixed carbon and the regeneration and uptake of inorganic nitrogen and phosphorus (Satinsky et al. 2017). These processes are further enhanced by the additional habitats and nutrient hotspots that the particles in the MTZ provide (Crump et al. 1999; Selak et al. 2022; Satinsky et al. 2017).

For more than 20 years, water quality and the impacts of anthropogenic activities in the Belgian part of the Schelde estuary (hereafter referred to as the Zeeschelde) have been monitored as a part of the OMES project (‘Onderzoek Milieu Effecten Sigmaplan’) (http://www.omes‐monitoring.be/en). Until the 2000s, strong eutrophication, with high ammonium and organic matter levels and low oxygen concentrations, was the main concern in the estuary (Cox et al. 2009). After water quality improvement in the first years of the 21st century, enhanced dredging activities to ensure the navigability of the channel and increased tidal pumping led to a sudden increase in suspended sediment concentrations, exacerbating primary production limitation in an already turbid system (Cox et al. 2019). These changes led to marked changes in microphytoplankton abundance and composition in the Zeeschelde (Amadei Martínez et al. 2023). De‐eutrophication, along with changes in sediment dynamics and discharge/precipitation, play a significant role in shaping the phytoplankton community composition. Furthermore, marked and long‐lasting shifts in phytoplankton community composition between 2002 and 2018 could be related to extreme precipitation events (Amadei Martínez et al. 2023).

Due to the limitations of morphology‐based plankton monitoring, there is limited information on the effects of environmental changes and the composition of pico‐ and nanoplankton in the Zeeschelde (Van Wichelen et al. 2006), with only a few studies addressing bacterial production (Goosen et al. 1997), abundance (Ouattara et al. 2013) and community composition (Bollmann and Laanbroek 2002). Similarly, few studies have focused on heterotrophic protists (Muylaert, Van Mieghem, et al. 2000). Consequently, there is a gap in our understanding of the relationships between phytoplankton, heterotrophic protists, and bacteria in the estuary.

In this study, we aim to provide a more comprehensive understanding of the spatiotemporal dynamics in the community composition of bacteria and microbial eukaryotes in the Zeeschelde using DNA‐amplicon sequencing data (16S‐ and 18S‐rRNA genes) between 2018 and 2021. We hypothesised that the turnover in microbial community composition in the estuary closely tracks the spatiotemporal dynamics of phytoplankton biomass, SPM and flushing events, and that these changes in community composition lead to changes in ecological functions delivered by microbiota.

## 2 Material and Methods

### 2.1 Data Collection

**FIGURE 1:**
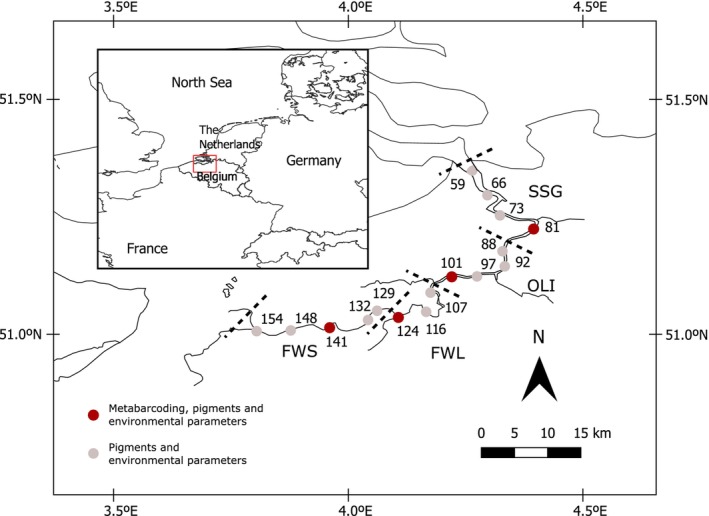
Monitoring stations in the Zeeschelde for the current study. The main stations of this study where metabarcoding, pigments and environmental parameters were measured are indicated in red. The station Antwerp (81 km distance from the mouth of the estuary at Vlissingen) belongs to the Strong Salinity Gradient zone (SSG), Temse (101 km) to the Oligohaline zone (OLI), Dendermonde (124 km) to the Freshwater Long Residence Time zone (FWL) and Uitbergen (141 km) to the Freshwater Short Residence Time zone (FWS) (Maris et al. 2014). In the rest of the stations, indicated in grey, only pigments and environmental parameters were measured, and these data were used only to visualise the environmental gradients in the contour plots.

From 2018 to 2021, subsurface water samples were collected monthly at different locations of the Zeeschelde estuary in Belgium. In the current study, we focused on the samples collected from March to October at four fixed stations in the estuary. Each station is in a different zone defined by salinity and residence time (Maris et al. 2014). In the freshwater zone with short residence time (FWS; chloride < 0.3 g L^−1^) we collected samples at Uitbergen (distance from the estuary mouth at Vlissingen: 141 Km), in the freshwater zone with long residence time (FWL; chloride < 0.3 g L^−1^) at Dendermonde (124 Km), in the oligohaline zone (OLI; chloride 0.3–3 g L^−1^) at Temse (101 Km) and in the strong salinity gradient zone (SSG; chloride 3–5.5 g L^−1^) at Antwerpen (81 Km) (Figure 1). Hereafter we refer to the stations using the abbreviations of each zone. Only to enhance the resolution in the contour plots of pigments and environmental parameters shown below, we also included data from the following stations: Melle (154 km), Wetteren (148 km), Appels (132 km), Sint Onolfs (129 km), Baasrode (116 km), Lippenbroek (107 km), Steendorp (97 km), Bazel (92 km), Kruibeke (88 km), Punt van Mesele (73 km), Liefkenshoek (66 km), and Greens (59 km). Environmental factors were measured using standard protocols described in Maris and Meire (2017). In brief, temperature and dissolved oxygen were measured using a WTW OXI 91 m, pH with a WTW pH 330 pH‐meter; total alkalinity was measured using automatic titration; CO_2_ was calculated from total alkalinity, pH, temperature and salinity using the CO2SYS software (Lewis and Wallace 2006); chloride, orthophosphate (soluble reactive phosphorus, SRP), ammonium, nitrate, nitrite, sulphate, total phosphorus (TP) and dissolved organic carbon (DOC) by segmented flow analysis. Particulate organic carbon (POC) was measured using an Elemental Analyser and suspended particulate matter (SPM) by gravimetry; dissolved silica (DSi) and biogenic silica (BSi) were measured using inductively‐coupled plasma atomic emission spectroscopy.

Data on total freshwater discharge were obtained at Schelle (93 Km) and Melle (154 Km) from the Hydraulics Information Center of Flanders Hydraulics Research. Mean discharge (Q*Q*) was calculated for each station for the 5 days before the sampling, using the values of Melle for the FWS and FWL and Schelle for the stations OLI and SSG.

Phytoplankton biomass was determined using pigment analysis, with Chlorophyll a (Chl a) as a proxy for algal biomass. Samples for pigment analysis were filtered over 25‐mm diameter Whatman GF/F glass fibre filters and analysed by High‐Performance Liquid Chromatography (HPLC) using the method of Van Heukelem and Thomas (2001). DNA samples were collected by vacuum filtering subsurface water on a 0.22 μm MF‐Millipore filter until the filter was saturated. The filter was immediately stored in liquid nitrogen and in the lab at −80°C until further analysis.

### 2.2 PCR and Illumina Sequencing

The DNA extraction was carried out with the DNeasy Powerlyzer Microbial Kit from Qiagen (Hilden, Germany). The polymerase chain reaction (PCR) amplification targeted the ribosomal RNA genes. For bacteria, the V1‐V3 regions of the 16S rRNA gene were amplified using pA (5′‐AGAGTTTGATCCTGGCTCAG‐3′) and BKL1 (5′‐GTATTACCGCGGCTGCTGGCA‐3′) primers (Tytgat et al. 2016). For eukaryotes, the V4 region was amplified using the TAReuk454FWD1 (5′‐CCAGCASCYGCGGTAATTCC‐3′) and the TAReukREV3 (5′‐ACTTTCGTTCTTGATYRA‐3′) primers (Stoeck et al. 2010). PCR and library preparation were done as in D'Hondt et al. (2018). Paired‐end (2 × 300 base pairs) sequencing was performed with the Illumina MiSeq technology (Illumina, San Diego, US) by Genewiz (Leipzig, Germany). For quality control, artificial mock communities, blanks and duplicate samples were included. Raw nucleotide sequences are available at the National Center for Biotechnology Information (NCBI) under BioProject PRJNA932988.

### 2.3 Data Analysis and Bioinformatics

We used the DADA2 (version 1.14.1) pipeline to process the amplicon sequences (Callahan et al. 2016). This workflow assigns sequencing data to amplicon sequence variants (ASVs) for each sample. Because for the 2018 samples the PCRs were carried out simultaneously for 16S and 18S genes, we split the fastq files per primer using an in‐house python script before running the reads in DADA2. Quality control, trim and filtering were done using the ‘filterAndTrim’ function. TrimLeft removed the primers and trimRight removed 10 bp at the end of each read. Afterwards, forward and reverse reads were truncated after 280 and 260 nucleotides in 16S and 250 and 220 in 18S respectively (truncLen). Sequences with expected errors (EE) higher than 5 and 6 in the forward and reverse reads, respectively, were removed in 16S and EE higher than 2 in 18S (maxEE). Sequences with ambiguities were removed (maxN). Pair ends were merged with a minimum overlap length of 12 bp, allowing for one mismatch using the function ‘mergePairs’. Each sequencing run (four in total) was processed in DADA2 separately. The different runs were merged by gene before removing the chimeras using the function ‘removeBimeraDenovo’. The 16S sequences were assigned to the SILVA database (Pruesse et al. 2007) version 138 using the dataset formatted by McLaren (2020) using the function ‘assignTaxonomy’. The 18S sequences were assigned to the Protist Ribosomal Reference database (PR^2^) (Guillou et al. 2013) version 4.14.0 (https://github.com/pr2database/pr2database/releases/tag/v4.14.0). The ASV table was further analysed using the ‘phyloseq’ package (version 1.36.0) (McMurdie and Holmes 2013). Contaminant sequences were removed following the method of Davis et al. (2018), removing 2% of reads in bacteria and 0.01% of reads in eukaryotes. Further, for the 16S gene, 40% of the reads, belonging to non‐identified taxa at phylum level, as well as chloroplast and mitochondrial sequences, were excluded from the analyses. For the 18S gene, 35% of the reads, belonging to non‐identified taxa at kingdom and infra‐kingdom level, as well as to the phylum Metazoa and the classes Embryophyceae and Streptophyta (in our dataset containing only vascular plants), were not included. For a subset of nine abundant unidentified eukaryotic ASVs at species level, manual annotations by online BLAST searches against the NCBI nt database were performed. Further, we replaced the annotation for the eukaryotic ASV20 from *Minidiscus spinulatus* to *Thalassiosira proschkinae*, based on (Muylaert and Sabbe 1996; Park et al. 2017). ASVs with a relative abundance lower than 1e^−5^% were omitted from the analyses. To predict the functions of bacteria, we use FAPROTAX (http://www.loucalab.com/archive/FAPROTAX/lib/php/index.php?section=Home). Ecological functions of eukaryotes were assigned using metaPR2 (version 1.0) (Vaulot et al. 2022).

### 2.4 Statistical Analysis

Statistical analyses were performed using R software (version 4.1.1; R Core Team 2021). Missing data for environmental parameters and Chl a were inferred with linear interpolation using the function ‘na_interpolation’ from the ‘imputeTS’ package (version 3.2) (Moritz and Bartz‐Beielstein 2017). The maximum percentage of interpolated data per parameter was 3%.

To explore changes in community composition, non‐metric multidimensional Scaling (NMDS) analyses, based on the Bray–Curtis dissimilarity matrix of the Hellinger transformed data, were performed using the ‘metaMDS’ function in ‘vegan’ (version 2.5.7; Oksanen et al. 2018). For this analysis, only ASVs with a relative abundance higher than 1% in at least 10 samples were considered. To assess the relationships between the environmental parameters and phytoplankton community structure, we used the ‘envfit’ function, which is a post hoc test that creates vectors that reflect the correlation between the NMDS axes and the variables of interest, in this case, the environmental parameters (Borcard et al. 2011). The significance level was calculated using 999 permutations and only the arrows belonging to significant parameters are shown in the plots. To test for differences in microbial community structure and ecological function related to distance from the mouth of the estuary, month, year and the interaction between month and year, we used PERMANOVAs using the ‘adonis2’ function in ‘vegan’.

Because the PERMANOVAs for microbial community composition revealed strong differences in dynamics between seasons and stations, we also analysed the datasets separately for each station, to focus on the seasonality. In addition, to visualise their seasonal turnover, the mean read proportion per month and station of the ASVs that significantly contributed to the NMDS per station, using the ‘envfit’ function, were plotted in a bubble plot. To explore the effects of the high precipitation events in July 2021 on microbial community composition and identify ASVs characteristic of such high precipitation events, we ran two additional NMDS analyses (one for bacteria and one for eukaryotes) without the March and April samples, as these presented a more homogeneous community composition along the longitudinal axis of the estuary due to higher early spring discharges. These analyses were also performed with the R package ‘vegan’. The significance threshold was *p* < 0.05 throughout the analyses.

## 3 Results

### 3.1 Variation in Environmental Conditions and Phytoplankton Biomass

**FIGURE 2:**
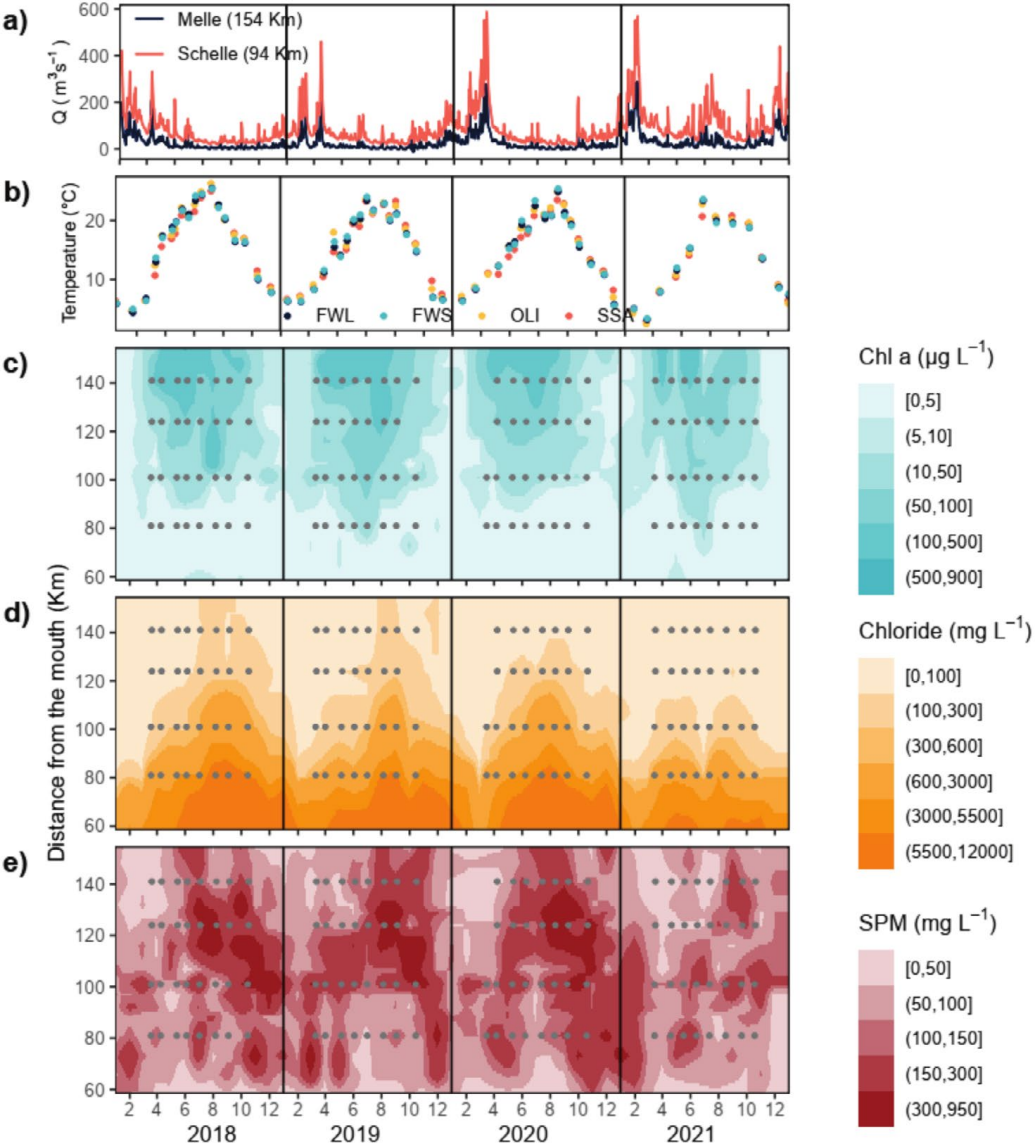
Spatiotemporal variability in the main abiotic and biotic parameters in the Zeeschelde from 2018 to 2021. (a) daily discharge values (Q) from the 2 discharge stations Melle and Schelle. (b) water temperature during sampling in FWS, FWL, OLI and SSG. (c–e) contour plots of Chl a, chloride and SPM. Grey dots indicate the dates and location at which DNA samples were collected in this study.

Daily discharge increased downstream and was typically higher in late winter–early spring, during periods of high precipitation (Figure 2a). Water temperature showed a strong seasonality, increasing from March to July/August and decreasing after August (Figure 2b). Chl a concentration showed a strong spatial and seasonal gradient, with annual mean Chl a concentration decreasing from FWS to SSG and with peak values occurring between May and August (Figure 2c). The auxiliary pigment ratio for diatoms (Fuco: Chl a) was slightly higher in spring/summer and lower in winter. Chlorophyte (Chl b: Chl a) and chlorophyte/cyanobacteria (Zea: Chl a) ratios were higher in winter, especially upstream, and lower in summer (Figure S1). Chloride concentration increased downstream and peaked during periods of low discharge in summer (Figure 2d) with upstream penetration of salt water as far as ~100 km in summer. The balance between freshwater discharge and tidal intrusion determines the seasonality in the position and extent of the MTZ (Cox et al. 2019), resulting in a winter maximum turbidity zone (MTZ) located around SSG and OLI, and a summer MTZ located between OLI and FWL. The summer MTZ was associated with lower dissolved oxygen concentration (Figure 2e; Figure S2a). CO_2_ concentrations were the lowest around the phytoplankton bloom, and downstream in SSG (Figure S2b). Concentrations of inorganic nutrients decreased downstream and were the lowest from May to September (Table S1).

The summer of 2021 was the wettest in Belgium since the start of meteorological records in 1833. This period of intense precipitation led to the highest summer discharges of this study period in July 2021, particularly downstream of Schelle, resulting in lower water temperatures (Figure 2b), a downstream shift of the chloride gradient by ~50 km (Figure 2d), a disruption of the freshwater summer MTZ (Figure 2e), and reduced Chl a concentration (Figure 2c). Chl a concentrations during the bloom at the station with the highest phytoplankton biomass (FWS) ranged from 186 to 251 μg L^−1^ between 2018 and 2020, dropping to 123 μg L^−1^ in 2021.

### 3.2 Spatiotemporal Changes in Microbial Community Composition

#### 3.2.1 Overall Composition of Bacterial and Eukaryote Biota

In total, 6.7 and 8.6 million raw reads were generated for 120 samples for the 16S rRNA barcode and for 118 samples for the 18S rRNA barcode, respectively. After filtering, denoising, merging, chimera removal, and downstream data cleaning steps, a total of 1.4 and 2.8 million reads were retained for the 16S and 18S rRNA barcode, respectively. The number of final sequenced reads per sample ranged between 2362 and 46,063 for the 16S and 2050–95,545 for the 18S rRNA barcode. This generated 7667 and 2863 unique ASVs for the 16S and 18S rRNA barcode, respectively. Most of the rarefaction curves for eukaryotes reached a plateau, suggesting that the sequencing depth was sufficient to represent the diversity of eukaryotic species. However, even though we found more ASVs for bacteria than for eukaryotes, the rarefaction curves suggest that bacterial diversity was only partially captured in this study, indicating a possible underestimation of the bacterial diversity in the current study (Figure S3).

In total, 28 phyla of bacteria and 29 phyla of eukaryotes were recorded. The phyla Actinomycetota (previously Actinobacteriota), Pseudomonadota (previously Proteobacteria) and Bacteroidota accounted for the majority of bacterial reads (47.0%, 22.5% and 19.5% respectively), with minor contributions from Nitrospirota (1.6%), Verrucomicrobiota (1.3%) and Cyanobacteria (1.3%). For eukaryotes, Ochrophyta and Ciliophora (42.8 and 17.9%, respectively) were the taxa with the highest number of reads, followed by Cryptophyta (8.3%), Cercozoa (6.6%), Fungi (5.4%), Dinoflagellata (4.7%), Chlorophyta (4.6%), Perkinsea (2.1%), Sagenista (1.3%) and Picozoa (1.3%). The remaining bacterial and eukaryotic groups represented less than 1% of the reads (Figure S4).

While most bacterial and eukaryote phyla were present along the entire estuarine gradient, the proportion of reads from Pseudomonadota increased downstream, as did the proportion of the mixo‐ and heterotrophic phyla of ciliates, cryptophytes, cercozoans, picozoa and dinoflagellates (Figure S5a,b). While for bacterial phyla, seasonal variation in relative abundance was less marked, eukaryote phyla showed distinct seasonal patterns, with Ochrophyta (dominated by diatoms), cryptophytes and ciliophora being present in March and April and mostly Ochrophyta being present at higher relative abundances between May and October, especially upstream (Figure S5b). Conversely, in the SSG zone, ciliates, cryptophytes, cercozoans and dinoflagellates were mainly present during summer, in relative abundances comparable to Ochrophyta. Picozoa and Perkinsea increased towards the end of the summer/early fall.

For bacteria, most ASVs were classified as minority ASVs, with relative abundances not exceeding 1% in at least 10 samples. In contrast, in the case of eukaryotes, a relatively small number of ASV's represented the majority of reads per sample (Figure S5a,b).

#### 3.2.2 Spatiotemporal Variation in Community Composition

We used PERMANOVA to explore major drivers of spatiotemporal turnover in the community composition of bacteria and microeukaryotes. Month (explaining 19% of the variance for bacteria and 17% for eukaryotes) and distance from the mouth of the estuary (contributing 15% for bacteria and 13% for eukaryotes) accounted for most of the variation in community composition at ASV level. The analysis further revealed significant but less pronounced interannual differences (2% for bacteria and 1% for eukaryotes) and interannual differences across seasons (2% for both bacteria and eukaryotes) in community turnover (Table S2).

**FIGURE 3:**
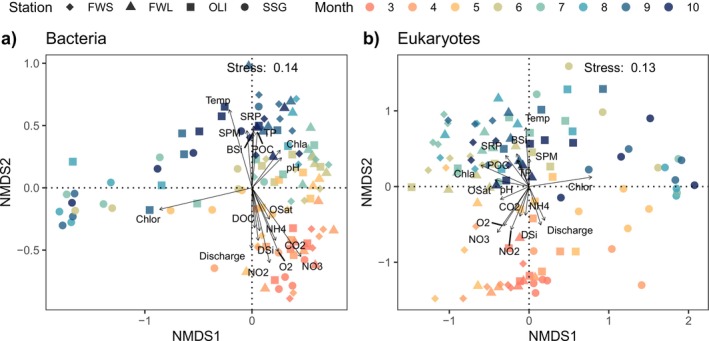
NMDS analyses of Hellinger transformed bacterial (a) and eukaryotic (b) abundance in the Zeeschelde (2018–2021). Colours indicate the sampling months and symbols the stations. The arrows represent variables that significantly (*p* < 0.05) contribute to explaining the variation in community structure according to the envfit test.

NMDS analysis showed that the community composition of bacteria and eukaryotes became more divergent between the zones as the season advanced (Figure 3a,b). From March to May, bacterial and eukaryotic assemblages were associated with higher discharge, N compounds, DSi, CO_2_ and oxygen; and for bacteria additionally also DOC. From May to July, bacteria and eukaryotes were associated with higher Chl a and pH, while from May to October they were associated with an increase of POC, TP, SRP, temperature, SPM and chloride (Figure 3a,b).

**FIGURE 4:**
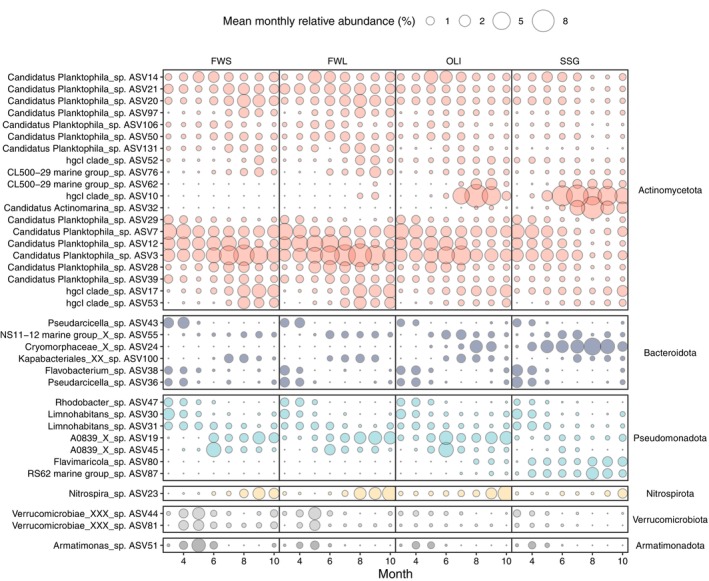
Bubble plot showing the mean monthly bacterial relative abundance per station for the ASVs that significantly contributed to the variation of the community composition in at least one of the NMDS analyses (Figure S6).

To explore seasonal turnover in community structure in more detail, we ran separate NMDS analyses for each station. These confirmed the strong seasonality in the microbial community (Figures S6 and S7). Some bacterial ASVs were significant contributors to the seasonality across all stations. In March and April, there was a higher relative abundance of *Flavobacterium* sp. (hereafter, ‘sp.’ and ‘spp.’ are used to denote one or multiple ASVs within the same genus, respectively) (Bacteroidota; ASV38), *Pseudarcicella* spp. (Bacteroidota; ASV36 and ASV43) and *Candidatus Planktophila* spp. (Actinomycetota; ASV29, and to a lesser degree also ASV7 and ASV12). From May to October, there was an increase of *hgcI clade* spp. (Actinomycetota; ASV17 and ASV53) and *Candidatus Planktophila* spp., with different ASVs belonging to these groups displaying different seasonal trends: for example, *Candidatus Planktophila* spp. ASV3 showed higher levels from March to August, ASV28 was more prevalent from May to August, and ASV20 increased from August to October (Figure 4; Figure S6).

Some bacterial ASVs were characteristic only in some stations. In late summer, there was an increase in the relative abundance of *Nitrospira* sp. (Nitrospirota), from FWS to OLI. Further, in FWS, a distinct succession occurred during the growing season, with *Rhodobacter* sp. (Pseudomonadota) and *Limnohabitans* spp. (Pseudomonadota; ASV30) characteristic for the period from March to May; *Verrucomicrobiae* spp. (Verrucomicrobiota; ASV44 and ASV81) and *Armatimonas* sp. (Armatimonadota) from April to June; and *Candidatus Planktophila* spp. (ASV21, ASV20, ASV97, ASV106, ASV50 and ASV131), *hgcl clade* spp. (ASV52), *CL500‐29 marine group* spp. (Actinomycetota; ASV76) and *A0839* spp. (Pseudomonadota; ASV19), from June to October. In FWL, bacterial seasonal turnover in community composition closely resembled that observed in FWS, except for *Candidatus Planktophila* spp. (ASV21, ASV97 and ASV106), which presented a significant seasonal turnover only in FWS, and *Candidatus Planktophila* sp. (ASV14) representative from May to July from FWL to SSG. In OLI, May to October was distinguished by an increase in *Candidatus Planktophila* sp. (ASV50), *A0839* spp. (ASV19 and ASV45), and *NS11‐12 marine group* sp. During the period from July to September, *CL500‐29 marine group* spp. (ASV62*), hgcl clade* spp. (ASV10), and *Cryomorphaceae* sp. (Bacteroidota) showed higher relative abundances. In SSG, March to April was characterised by a higher relative abundance of *Limnohabitans* spp. (ASV31 and ASV30) and *Pseudarcicella* spp. (Bacteroidota; ASV43); from May onwards, *Candidatus Actinomarina* sp., *CL500‐29 marine group* spp. (ASV76), *Kapabacteriales* sp. (Bacteroidota), *Flavimaricola* sp. (Pseudomonadota) and *RS62 marine group* sp. (Pseudomonadota) became more abundant. The trends for ASVs from the *hgcl clade* spp. (ASV10), *CL500‐29 marine group* spp. (ASV62), Cryomorphaceae sp., and NS11‐12 marine group sp. (Bacteroidota) were very similar to those observed in OLI (Figure 4; Figure S6).

In the case of eukaryotic phototrophs, some ASVs were important contributors to the seasonality in all the stations (Figure S7). *Cryptomonas curvata* and *C. marssonii* (Cryptophyta) were most abundant from March to June while the diatom *Thalassiosira allenii* was more characteristic from August onwards.

**FIGURE 5:**
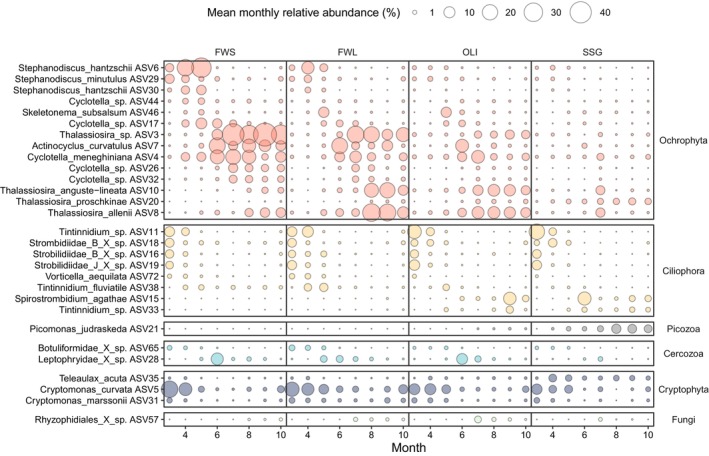
Bubble plot showing mean monthly eukaryotic relative abundance per station for the ASVs that significantly contributed to the variation of the community composition in at least one of the NMDS analyses (Figure S7).

All the eukaryotic phototrophs representative at different moments of the season were diatoms (Ochrophyta), except for the cryptophyte *Teleaulax acuta* which was characteristic from June to October in SSG. From FWS to OLI, the diatoms *Stephanodiscus hantzschii* (ASV6) and *Stephanodiscus minutulus* were characteristic from March to May while *Thalassiosira* sp. was representative from July onwards. These taxa presented decreasing relative abundance downstream. Further, diatoms in FWS were distinguished by a higher relative abundance of *Stephanodiscus hantzschii* (ASV30) and *Cyclotella* spp. (ASV44) from March to May, and from June to October, by *Actinocyclus curvatulus* , *Cyclotella meneghiniana* and *Cyclotella* spp. (ASV26 and ASV32). In FWL, March–May was characterised by *Skeletonema subsalsum* . May–June was marked by *Cyclotella* sp. (ASV17). From July onwards, there was an increase in *Thalassiosira anguste‐lineata*. In OLI, March to May was characterised by ASVs found also upstream and, June to August was distinguished by an increase of *Actinocyclus curvatulus, Cyclotella* spp. (ASV17). Furthermore, from June to October, *Thalassiosira anguste‐lineata* and *Cyclotella meneghiniana* were at higher relative abundances. ASVs characteristic of SSG from March to May were also present in the rest of the stations. Around July, the relative abundance of the diatom *T. anguste‐lineata* was at its highest. From June to October, the relative abundance of *Cyclotella meneghiniana* and *Thalassiosira proschkinae* increased (Figure 5; Figure S7).

Eukaryotic heterotrophs showing pronounced seasonality in all the stations include *Tintinnidum* sp., Strombidiidae *B* spp. (ASV18 and ASV16) and Strombidiidae *J* sp. (Ciliophora), and were most important from March to June. Regarding representative taxa only in some stations, in FWS and FWL during March–May, there was a higher relative abundance of *Vorticella aequilata* (Ciliophora) and Botuliformidae (Cercozoa). Further, in FWL, May–June was characterised by Leptophryidae (Cercozoa). *Tintinnidum fluviatile* (Ciliophora) was found at higher relative abundances from FWL to OLI between March and May. In OLI, June to August was characterised by an increase in Leptophryidae. In September and October, the relative abundance of *Spirostrombidium agathae* and *Tintinnidium* spp. (ASV33) increased. In SSG, around July, the relative abundance of *Rhyzophidiales* sp. (Fungi) was at its highest. From June to October, the relative abundance of *S. agathae*, *Tintinnidium* spp. (ASV33) and *Picomonas jusraskeda* (Picozoa) increased (Figure 5; Figure S7).

#### 3.2.3 Trends in Functional Group Composition

**FIGURE 6:**
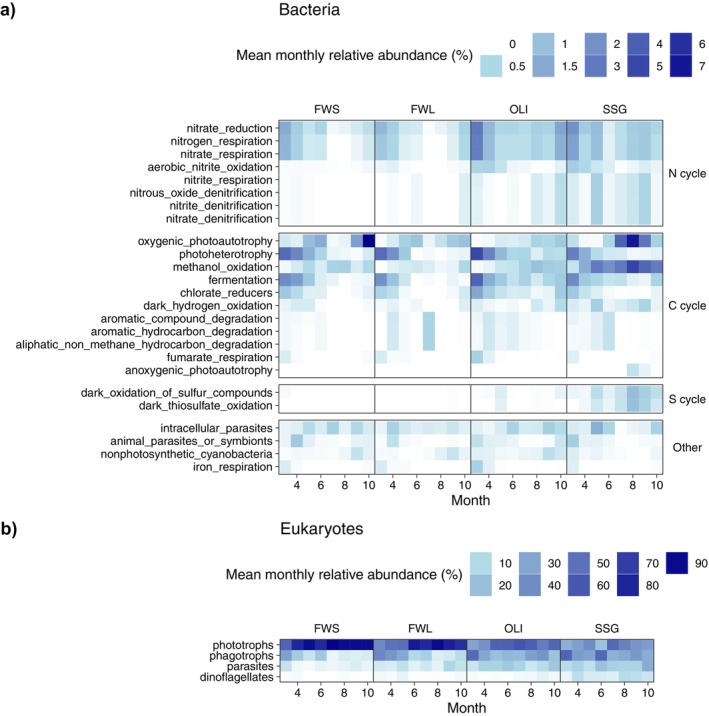
Seasonal changes in the mean proportion of reads (4 years) assigned to ecological functions of (a) bacteria using FaProTax and (b) eukaryotes using metaPR2 in the four zones. Note that the same colour in the two plots corresponds to a different value.

Functional assignments for the majority of bacteria remain unknown as only 9% of the reads could be successfully annotated using FAPROTAX. From this small proportion of functionally annotated ASVs, functional groups related to the carbon cycle, including oxygenic photoautotrophy, photoheterotrophy, methanol oxidation, and fermentation, had the highest average relative abundance (1.1%, 1.0%, 0.9%, and 0.7%, respectively), followed by those related to the nitrogen cycle, including nitrate reduction, nitrate respiration, and nitrogen respiration (0.7%, 0.6%, and 0.6%, respectively). PERMANOVA analysis suggested that the predominant factors influencing variation in these bacterial functions were months, distance from the mouth of the estuary, and, to a lesser extent, year (Table S3). Figure 6a shows that the functional groups such as photoheterotrophy, fermentation, nitrate reduction, nitrogen respiration, and nitrate respiration were relatively more important in March and April in all stations. In contrast, oxygenic photoautotrophy was more important in SSG and FWS in the second half of the year. In addition, methanol oxidation was more pronounced from May to October, especially in SSG. Furthermore, Figure 6a also suggests that the functions involved in the nitrogen cycle, hydrocarbon degradation, and the sulfur cycle were more important downstream, in SSG and OLI. Some functions displayed distinct interannual variability, including photoheterotrophy, oxygenic photoautotrophy, methanol oxidation, fermentation, nitrate reduction, nitrate respiration, and nitrogen respiration (Figure S8).

Using metaPR2, we could assign an ecological function to 95% of the eukaryotic reads of our dataset. Phototrophs were the most important functional group (mean proportion of reads 59.4%), followed by phagotrophs (23.9%), parasites (8.9%) and dinoflagellates (2.4%). Dinoflagellates were considered a separate functional group because of their large variability in ecological functions (phototrophy, heterotrophy or mixotrophy) (Mordret et al. 2018) and because they were most important in SSG. PERMANOVA analysis suggested that the predominant factors influencing the variation in eukaryotic ecological functions were month and distance from the mouth of the estuary (Table S3). The mean relative abundance of phototrophs was lower in April and May in all stations and overall decreased downstream. The mean relative abundance of phagotrophs, parasites, and dinoflagellates increased downstream. In addition, the relative abundance of phagotrophs was higher during the first half of the year but lacked a clear seasonality in OLI and SSG. Similarly, the relative abundance of parasites did not present a clear seasonal trend, while dinoflagellates were more abundant from August to October (Figure 6b).

#### 3.2.4 Effect of a Summer High Precipitation Event on the Community Composition of the Zeeschelde

The microbial community composition in the Zeeschelde showed interannual differences in seasonality (cf. significant interaction between year and month, PERMANOVA, Table S2). We therefore ran a separate NMDS, with only the samples that presented the highest interannual variability (May‐Oct), to assess the effect of the high precipitation event in July 2021 on microbial community composition. The NMDS analyses of both bacteria and eukaryotes showed that the community composition presented less spatial variability in 2021 compared to the other years (except for eukaryotes in SSG) (Figure S9), coinciding with a less steep chloride gradient in 2021. In all stations, there was a higher abundance of Actinomycetota *Candidatus Planktophila* spp. (ASV21, ASV20, ASV3, ASV7, and ASV28), and lower abundance of Actinomycetota *CL500‐29 marine group* spp. (ASV62), *hgcI clade* spp. (ASV10), Candidatus *Actinomarina* sp., Pseudomonadota *Flavimaricola* sp., *RS62 marine group* sp., and Bacteroidota *Cryomorphaceae* sp. (Figure S9). In eukaryotes, from FWS to OLI, 2021 was associated with a higher relative abundance of diatoms, including *Cyclotella meneghiniana, Actinocyclus curvatulus, Cyclotella* spp. (ASV26 and ASV32) and *Thalassiosira* sp. The eukaryotic community composition of SSG in 2021 was similar to previous years (Figure S9).

## 4 Discussion

In the freshwater and brackish tidal Zeeschelde estuary, bacterial and microbial eukaryotes display pronounced, annually recurring, spatial and temporal turnover patterns in taxonomic and functional community structure. These are largely driven by the balance between freshwater discharge and tidal incursion. In winter and early spring, high freshwater discharge shifts the MTZ further downstream to the OLI and SSG zones, coinciding with the lowest phytoplankton biomass. With increasing light and temperature, phytoplankton biomass increases, resulting in an extended bloom between May and August, especially in the more upstream regions of the estuary. In summer, reduced discharge allows deeper saltwater intrusion in the estuary, resulting in a steeper salinity gradient and an upstream shift in the position of the MTZ to the FWL zone. Our study adds to previous research that has highlighted the importance of the discharge‐driven temporal dynamics of the estuarine salinity gradient in controlling essential processes in the estuary, such as sediment dynamics (Burchard et al. 2018; Cox et al. 2019), biogeochemical cycling (Regnier et al. 2013; Rios‐Yunes et al. 2023) and the distribution and activity of microorganisms (Muylaert et al. 2009; Amadei Martínez et al. 2023; this study).

### 4.1 Seasonal Turnover in Microbial Community Composition

The results of the NMDS analyses revealed that microbial community composition varied along the salinity gradient and displayed marked seasonal changes in ASV composition. For eukaryotic phototrophs, from March to April, a more uniform community composition occurs along the estuarine gradient, with the most characteristic eukaryotic phototrophs being cryptophytes and diatoms, both previously reported as common spring taxa in the most upstream, freshwater reaches of the estuary (Muylaert, Sabbe, et al. 2000). From May onwards, as a steeper salinity gradient develops, we observe a greater divergence in community composition between the stations. Several freshwater taxa, such as one ASV of *Thalassiosira* sp., *Actinocyclus curvatulus* and five different *Cyclotella* ASVs, occurred at higher relative abundance upstream. Fewer ASVs were more abundant in the freshwater/brackish water transition zone, including *Thalassiosira alleni*, *T. anguste‐lineata* and *Skeletonema subsalsum* . Only the brackish diatom *Minidiscus* (formerly *Thalassiosira*) *proschkinae* presented higher relative abundances downstream, in agreement with its occurrence reported in (Muylaert and Sabbe 1996; Muylaert, Sabbe, et al. 2000). The end of the growing season was characterised by a persistent higher presence of brackish/marine taxa from the FWL to the SSG zone, a slight decrease in *Cyclotella* ASVs and an increase in *Thalassiosira* ASVs.

From March to April, characteristic bacterial taxa included the photoheterotrophic *Limnohabitans* (Pseudomonadota) (Kasalický et al. 2018); the chemoheterotrophic *Pseudarciella* (Bacteroidota) (Cruaud et al. 2020), and *Armatimonas* (Armatimonadota) (Tamaki et al. 2011), *Verrucomicrobiae* (Verrucomicrobiota), *Flavobacterium* (Bacteroidota) and *Rhodobacter* (Pseudomonadota), all of which have previously been reported to be associated with phytoplankton (Teeling et al. 2012; Taylor et al. 2014; Laas et al. 2022), and are known to consume algal polymers (Kirchman 2002; Taylor et al. 2014; Hugerth et al. 2015). During the warmer months, the Actinomycetota *Candidatus Planktophila* was one of the most common bacterial taxa, represented by different ASVs, especially upstream. *Candidatus Planktophila* represents a largely uncultured group of bacteria, most likely because of their high degree of metabolic dependency on co‐existing microbes (Jezbera et al. 2009). This group of photoheterotrophic bacteria is one of the most prevalent in freshwater ecosystems, and probably plays an important role in degrading the complex mixture of organic matter derived from plant biomass (Ghai et al. 2014; Neuenschwander et al. 2018), making them good candidates to be key players in the decomposition of organic matter in the Zeeschelde. Downstream, in SSG, we found several taxa that are mainly known from marine habitats, such as the Bacteroidota *NS11‐12 marine group* sp., Cryomorphaceae, Candidatus *Actinomarina* sp., RS62 marine group sp. and *Flavimaricola* sp. (Ghai et al. 2013; Coclet et al. 2019; Wirth et al., Wirth and Whitman 2018; Bowman 2020). After the phytoplankton bloom, there was an increase in the relative abundance of the nitrifier *Nitrospira* sp. (Daims et al. 2015) and of *hgcl clade* sp. which can reduce nitrate and phosphate (Liu et al. 2020). This may suggest seasonality in the nitrogen cycle as has already been described in benthic habitats of the estuary, with higher denitrification rates at the end of the growing season when elevated temperature, lower oxygen and higher turbidity favour anoxic reactions (Rios‐Yunes et al. 2023). Our FAPROTAX analysis further suggests a downstream increase in anaerobic processes. Because the oxygen concentration measured in the water column during this study was never < 4.6 mg L^−1^, our results suggest that these anaerobic processes could be performed by particle‐attached bacteria in anoxic micro‐zones of particle flocs (Selak et al. 2022).

Among heterotrophic eukaryotes, phagotrophs were present throughout the growing season, while parasites became more important towards autumn. Ciliates (phagotrophs) were most abundant during March–April. Muylaert, Van Mieghem, et al. (2000), using microscopy counts, found a similar trend, which attributed the higher abundance of ciliates in winter compared to summer due to release from top‐down control by rotifers. From May onwards, ciliates became slightly less important, especially in the SSG zone, and *Picomonas judraskeda* increased, which is a widespread marine picozoan that uses endocytosis to consume nano‐sized organic matter (Seenivasan et al. 2013; Schön et al. 2021). Interestingly, chytrids of the order of *Rhyzophidiales* were found at higher relative abundances during and after the peak of the phytoplankton bloom. Chytrids infect phytoplankton cells, thereby controlling phytoplankton populations (Frenken et al. 2016). Additionally, we detected the presence of Perkinsea at the end of the summer, although their relative abundance was > 10% in only 3 samples. Perkinsea is a very diverse and largely understudied parasitic taxon, mostly described as parasite of marine dinoflagellates, molluscs, and fish (Itoïz et al. 2022). Their role in ecosystem functioning remains largely unknown (Jephcott et al. 2016). Our data indicate that chytrids and Perkinsea could be involved in phytoplankton bloom termination in the Zeeschelde.

Our study adds new insights into the spatiotemporal dynamics of bacteria and heterotrophic micro‐eukaryotes, complementing earlier studies on phytoplankton dynamics in the Zeeschelde based on morphological species taxonomy (Muylaert, Sabbe, et al. 2000; Muylaert et al. 2001, 2009; Muylaert and Vyverman 2006; Amadei Martínez et al. 2023). While in general the majority of phyla identified using morphology‐based methods were also found in the molecular inventory of this study, the V4‐18S marker also captured the taxonomic diversity of understudied or morphologically difficult‐to‐identify taxa, including for example, ciliates, cercozoan and fungi (Figure S10). In addition, metabarcoding revealed previously undetected diversity in certain phototrophic taxa, suggesting that some species delineated based on morphology (Muylaert et al. 2009; Amadei Martínez et al. 2023) represent complexes of closely related (semi)cryptic species. Interestingly, certain *Cyclotella* ASVs exhibited distinct seasonal distributions, suggesting that these (semi)cryptic taxa have different ecological niches. Surprisingly, even though we found 13 and 55 different genera of Cyanobacteria and Chlorophyta, respectively, in our metabarcoding dataset, their relative abundances were very low, while we were able to detect their auxiliary pigments in significant quantities. This suggests a possible bias towards the quantification of those taxa in our methodology that needs to be further explored. One possible explanation would be that the primers used did not capture these groups very well (van der Loos and Nijland 2021), but other studies using the same primers did detect high abundances of both groups (Tanttu et al. 2023). In addition, it is known that some eukaryotic taxa have higher copy numbers of the 18S rRNA gene, such as ciliates and dinoflagellates, leading to an overestimation of their importance (Gong et al. 2013; Jiang et al. 2022).

### 4.2 Microbial Functional Group Turnover in Relation to the MTZ

Our study shows that the development of the spring–summer phytoplankton bloom, within the freshwater tidal zones (FWS and FWL), and its interaction with the summer MTZ in the FWL zone, contributed to marked changes in the functional composition of the microbial communities. Upstream of the MTZ, the eukaryotic communities were dominated by phototrophs, whereas downstream of this zone, the relative abundance of phototrophic eukaryotes decreased, and the importance of phagotrophs (mostly ciliates and cercozoa) and parasites (dominated by fungi and perkinsoids) increased. This suggests that, as freshwater phytoplankton reaches the summer MTZ, lower light conditions limit further growth and induce stress, allowing eukaryotic heterotrophs to graze on or infect the phytoplankton, increasing their relative abundance. Microzooplankton can remove up to 80% of the phytoplankton standing stock in the estuary (Lionard et al. 2005), resulting in the colonisation of the remaining algal necromass by bacteria and contributing to the decomposition and recycling of organic matter (Wang et al. 2024). The importance of the MTZ for promoting heterotrophic processes was further supported by the decrease of the oxygen saturation levels and increase of CO_2_ around the summer MTZ, and the higher relative abundance of bacteria that decompose organic matter (e.g., *Candidatus Planktophila*) in that zone. These observations corroborate previous reports of low primary production and high bacterial production in the MTZ compared to the rest of the estuary (Goosen et al. 1997).

### 4.3 Effects of an Extreme Summer Precipitation Event on Microbial Community Composition

In mid‐July 2021, Belgium experienced unprecedented high precipitation within a 48‐h period. This extreme event led to severe floods in the southern part of the country (Journée et al. 2023). In the Zeeschelde, it resulted in peaks in the discharge, and import of organic matter, SPM and nutrients from the tributaries (mostly the river Rupel), leading to high N concentrations and the lowest oxygen concentrations measured during this study, and a less pronounced summer salinity and SPM gradient. We expected this event to have a profound effect on plankton community composition, as had been reported for previous flushing events in the Zeeschelde (Muylaert et al. 2001; Muylaert and Vyverman 2006; Amadei Martínez et al. 2023). Our study shows that the heavy rains in July 2021 resulted in a decrease in residence time and dilution of the phytoplankton bloom, dampening its development and/or accumulation, a downstream displacement of freshwater estuarine species (e.g., *Candidatus Planktophila* sp., *Cyclotella meneghiniana* , *Actinocyclus curvatulus* , and *Cryptomonas curvata* ), and a decrease in brackish/polyhaline species (e.g., *CL500‐29 marine group* sp., *hgcI clade* sp., *Actinomarina* sp., *Thalassiosira proschkinae, and Picomonas judraskeda*). Flushing events thus disrupt the gradient in the ecological functions, possibly altering the normal filtering functions of the ecosystem at that time of the year (Paerl et al. 2014), which may have implications on the estuarine ecosystem but also in adjacent coastal areas (Kruk et al. 2021).

## Author Contributions

L.A.M.: conceptualisation, formal analysis, data curation, visualisation and writing – review and editing. K.S.: conceptualisation, supervision, project administration, writing – review and editing. S.D., R.D., I.D., T.V., P.C. and N.B.: investigation. T.M.: data curation, investigation and project administration. P.M.: project administration and funding acquisition. W.V.: conceptualisation, writing – review and editing, supervision, project administration and funding acquisition.

## Conflicts of Interest

The authors declare no conflicts of interest.

## Supporting information


**Data S1.**emi470135‐sup‐0001‐Supinfo.

## Data Availability

The data that support the findings of this study are available from the corresponding author upon reasonable request.
