# Supplementary material for: Freshwater Discharge and Salinity Drive Taxonomic and Functional Turnover of Microbial Communities in a Turbid Macrotidal Estuary
Source: Environ Microbiol Rep. 2025 Jul 14;17(4):e70135. doi: 10.1111/1758-2229.70135 (PMC12256932; doi:10.1111/1758-2229.70135)
Supplement: Supplementary file 1 — Data S1.emi470135‐sup‐0001‐Supinfo. [file EMI4-17-e70135-s001.docx]

## Appendix

**Freshwater discharge and salinity drive taxonomic and functional turnover of microbial communities in a turbid macrotidal estuary**

Luz Amadei Martínez ^1, ⁎^ (<https://orcid.org/0000-0001-5960-7972> ), Koen Sabbe ^1^ (<https://orcid.org/0000-0001-5163-5581> ), Sofie D'hondt ^1^ (<https://orcid.org/0000-0002-2128-0553> ), Renaat Dasseville ^1^ (<https://orcid.org/0000-0003-3399-2651> ), Ilse Daveloose ^1^, Tine Verstraete ^1^, Peter Chaerle ^1^ (<https://orcid.org/0000-0002-8216-0299> ), Natacha Brion ^2^ (<https://orcid.org/0000-0002-3021-1445>), Tom Maris ^3^ (<https://orcid.org/0000-0002-9819-6771> ), Patrick Meire ^3^ (<https://orcid.org/0000-0003-2599-5350> ) & Wim Vyverman ^1,*^ (<https://orcid.org/0000-0003-0850-2569> )

^1^ Laboratory of Protistology and Aquatic Ecology, Department of Biology, Ghent University, Krijgslaan 281-S8, 9000 Ghent, Belgium

^2^ Department of Archeology, Environmental change and Geochemistry, Vrije Universiteit Brussel, Pleinlaan 2, 1050 Brussels, Belgium

^3^ ECOSPHERE Research Group, University of Antwerp, Universiteitsplein 1C, 2610 Wilrijk, Belgium

^⁎^ Correspondence: [luz.amadeimartinez@ugent.be](mailto:luz.amadeimartinez@ugent.be) & [Wim.Vyverman@ugent.be](mailto:Wim.Vyverman@ugent.be)

| 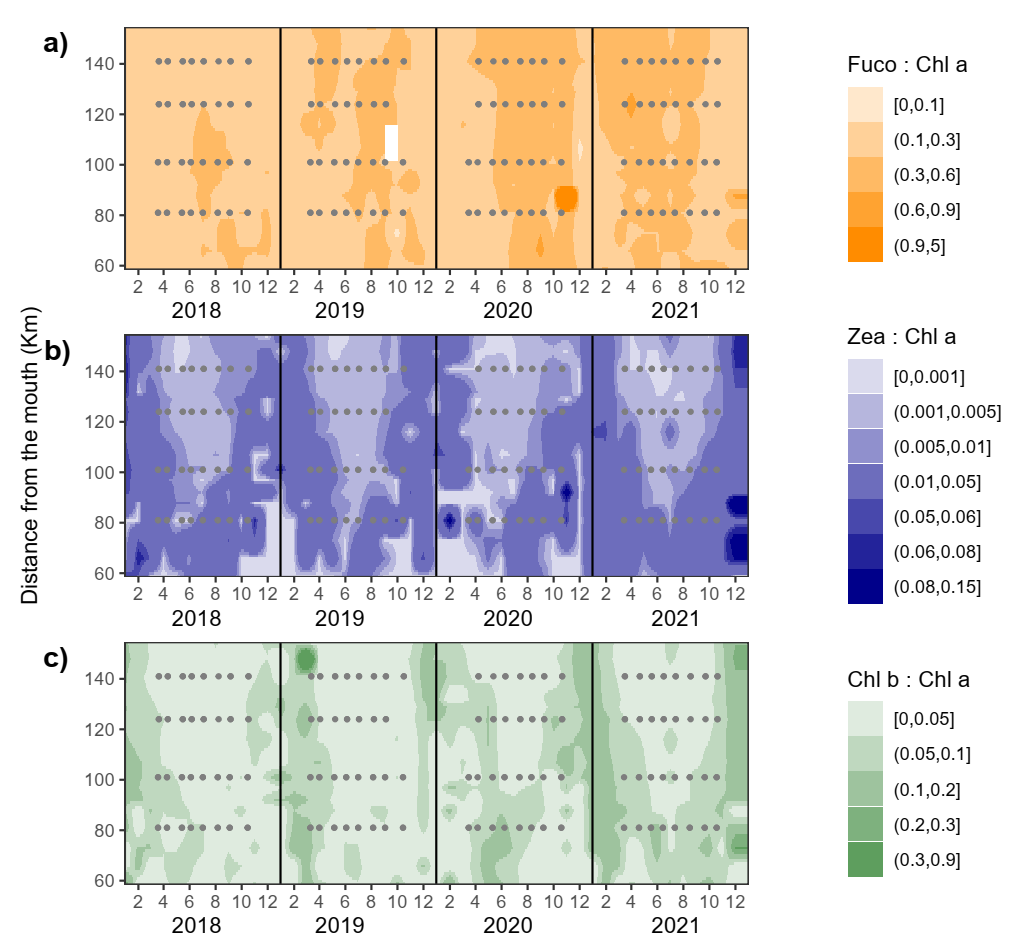 |
| --- |
| Figure S1. Spatio–temporal variability in a) fucoxanthin, b) zeaxanthin and c) Chl b in the Zeeschelde from 2018 to 2021. The grey dots indicate the date at which DNA samples were collected for this study. We use these auxiliary pigments as a proxy of the presence of diatoms (Fuco : Chl a), b) Cyanobacteria (Zea : Chl a) and c) Chlorophytes (Chl b : Chl a). |

| 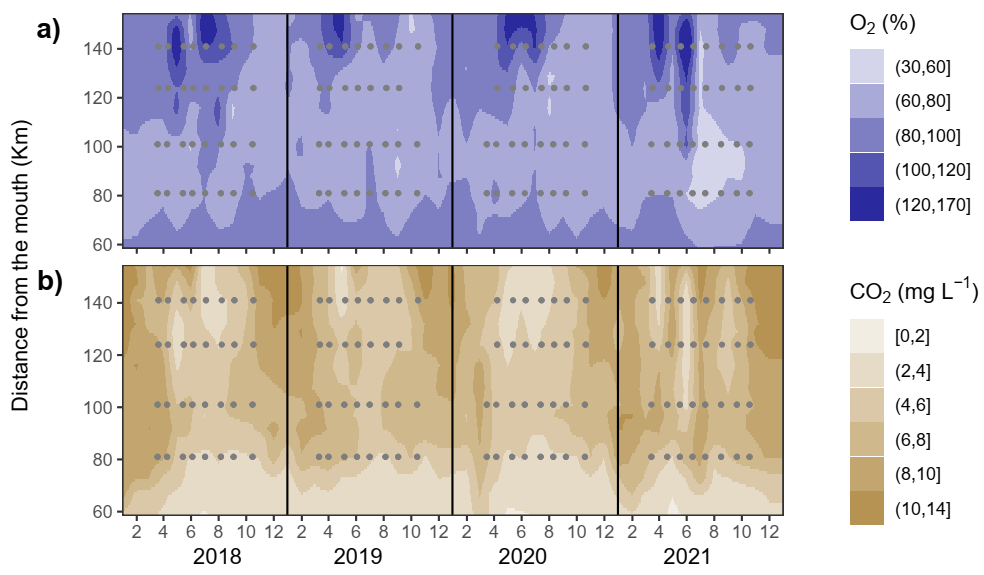 |
| --- |
| Figure S2. Spatio–temporal variability in a) O_2_ and b) CO_2_ in the Zeeschelde from 2018 to 2021. The grey dots indicate the date at which DNA samples were collected for this study. |

| 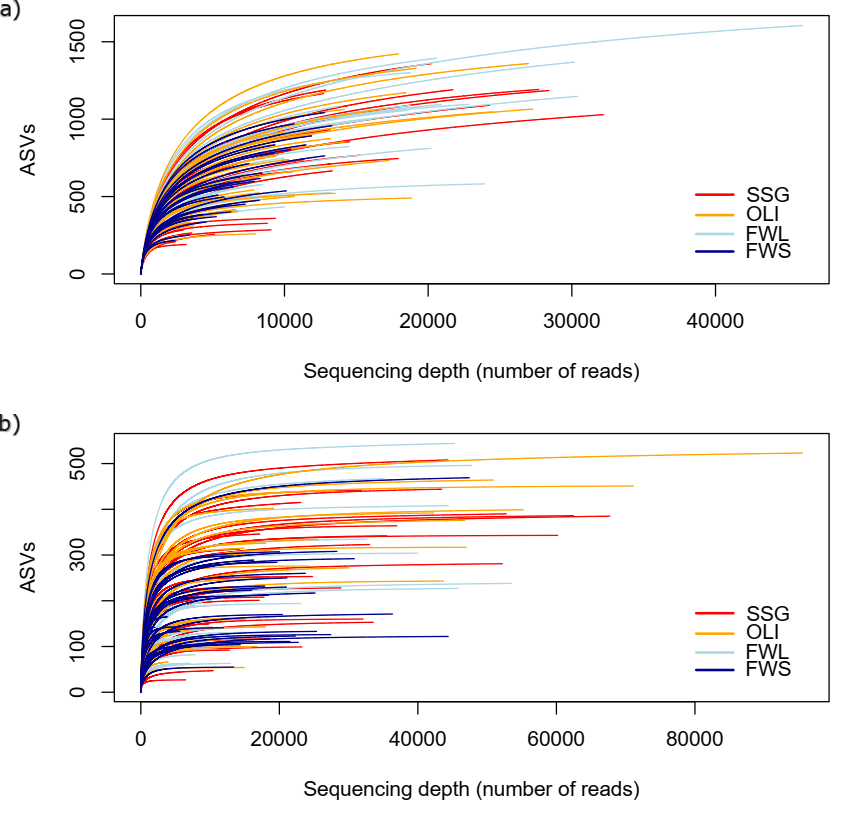 |
| --- |
| Figure S3. Rarefaction curves for the selected samples for this study. a) Bacteria. b) Eukaryotes. |
| 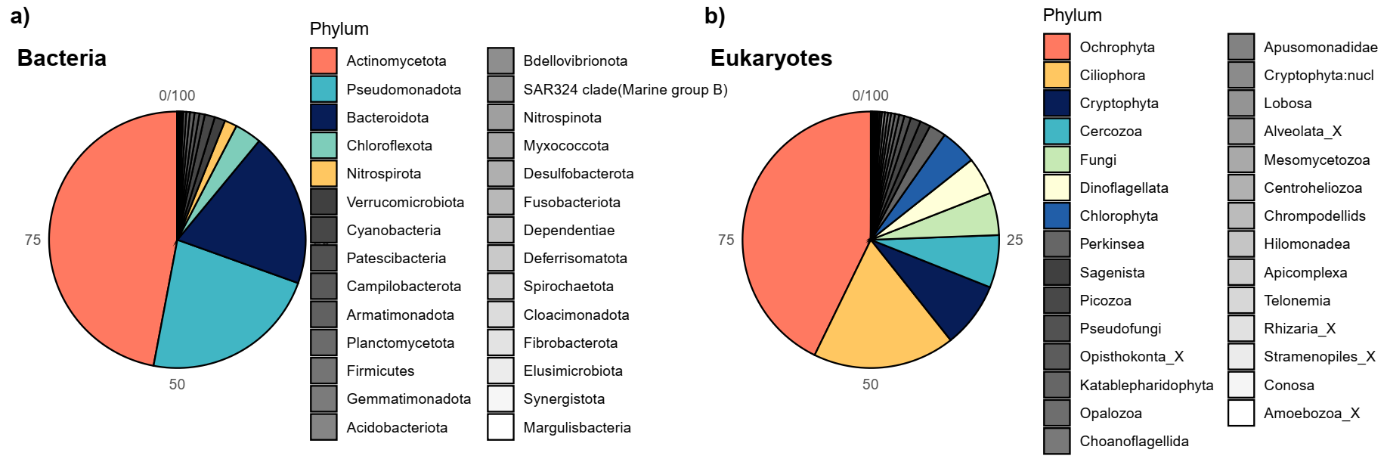 |
| *Figure S4. Summary of the percentage of reads per phylum for the whole data set. a) Bacteria. b) Eukaryotes. Taxa with reads relative abundance < 1% are listed in grey.* |

| Table S1. Summary of the environmental parameters in four stations of the Zeeschelde. Abbreviations: Standard deviation (SD); Minimum (Min); Maximum (Max); Suspended Particulate Matter (SPM); Particulate Organic Matter (POC); Dissolved Organic Carbon (DOC); Temperature (Temp); dissolved silica (DSi) and biogenic silica (BSi); Soluble Reactive Phosphorus (SRP); and Total Phosphorus (TP). |
| --- |
| \|  \|  \| **FWS** \| \| \| **FWL** \| \| \| **OLI** \| \| \| **SSG** \| \| \| \| --- \| --- \| --- \| --- \| --- \| --- \| --- \| --- \| --- \| --- \| --- \| --- \| --- \| --- \| \| **Parameter** \| **Units** \| **Mean ± SD** \| **Min** \| **Max** \| **Mean ± SD** \| **Min** \| **Max** \| **Mean ± SD** \| **Min** \| **Max** \| **Mean ± SD** \| **Min** \| **Max** \| \| **Discharge** \| m³s-1 \| 12.4 ± 13.2 \| 0 \| 56.6 \| 12.5 ± 13.3 \| 0 \| 56.6 \| 19.7 ± 36 \| 0.2 \| 192.4 \| 71.3 ± 77.9 \| 20.3 \| 444.8 \| \| **Chl a** \| µg L^-1^ \| 72 ± 52.9 \| 6.7 \| 251.9 \| 40.7 ± 28.4 \| 4.9 \| 102 \| 18.1 ± 18.6 \| 2.8 \| 87.1 \| 3.8 ± 4.4 \| 1.3 \| 25.6 \| \| **Chloride** \| mg L^-1^ \| 80.4 ± 17.3 \| 55 \| 120 \| 105 ± 49.3 \| 53.8 \| 243 \| 498.5 ± 624.6 \| 36.6 \| 2260 \| 2707 ± 2536.9 \| 118.7 \| 7800 \| \| **SPM** \| mg L^-1^ \| 102 ± 93 \| 20 \| 423 \| 133.7 ± 103.2 \| 36 \| 483 \| 180 ± 105.2 \| 39.2 \| 519 \| 103.2 ± 63.2 \| 27.4 \| 279.6 \| \| **POC** \| mg L^-1^ \| 5.2 ± 3.1 \| 1.7 \| 13.8 \| 5.4 ± 3.3 \| 2.0 \| 14.6 \| 5.8 ± 2.9 \| 1.4 \| 14.0 \| 3.8 ± 2.9 \| 1.2 \| 16.5 \| \| **DOC** \| mg L^-1^ \| 4.8 ± 0.8 \| 3.7 \| 7.2 \| 5.1 ± 1.1 \| 3.9 \| 8.9 \| 5.1 ± 0.8 \| 3.9 \| 7.2 \| 5.1 ± 0.8 \| 3.5 \| 6.7 \| \| **CO_2_** \| mg L^-1^ \| 5.5 ± 2.6 \| 1.9 \| 10.2 \| 5.9 ± 1.9 \| 2.4 \| 9.5 \| 6.6 ± 1.7 \| 3.2 \| 9.8 \| 5.4 ± 1.9 \| 2.7 \| 8.9 \| \| **Temp** \| °C \| 17.4 ± 5.2 \| 6.4 \| 25.4 \| 17.3 ± 5.2 \| 6.8 \| 25.5 \| 17.1 ± 5.4 \| 6.4 \| 26.3 \| 16.8 ± 5.2 \| 6.5 \| 25 \| \| **DSi** \| mg L^-1^ \| 5.5 ± 5.4 \| 0.1 \| 14 \| 5.7 ± 4.7 \| 0.3 \| 13.5 \| 7.6 ± 3.9 \| 1.4 \| 13.4 \| 8.4 ± 3.5 \| 2.2 \| 14.7 \| \| **BSi** \| mg L^-1^ \| 2.5 ± 2.3 \| 0 \| 8.5 \| 2.5 ± 2.1 \| 0 \| 7 \| 2.1 ± 1.6 \| 0 \| 5.4 \| 1.1 ± 0.8 \| 0 \| 2.7 \| \| **NH_4_ ^+^** \| mg L^-1^ \| 0.1 ± 0.2 \| 0 \| 0.6 \| 0.1 ± 0.2 \| 0 \| 1.2 \| 0.1 ± 0.1 \| 0 \| 0.4 \| 0.1 ± 0.1 \| 0 \| 0.4 \| \| **NO_3_ ^-^** \| mg L^-1^ \| 4.4 ± 1 \| 2.7 \| 6.6 \| 4.1 ± 1 \| 2.5 \| 6.4 \| 4 ± 0.9 \| 2.8 \| 6.1 \| 3.5 ± 1.1 \| 0.7 \| 5.7 \| \| **NO_2_ ^-^** \| mg L^-1^ \| 0.1 ± 0.1 \| 0 \| 0.2 \| 0 ± 0 \| 0 \| 0.2 \| 0 ± 0 \| 0 \| 0.1 \| 0 ± 0 \| 0 \| 0.1 \| \| **SRP** \| mg L^-1^ \| 0.2 ± 0.1 \| 0.1 \| 0.3 \| 0.2 ± 0 \| 0.1 \| 0.3 \| 0.2 ± 0 \| 0.1 \| 0.2 \| 0.1 ± 0 \| 0 \| 0.2 \| \| **TP** \| mg L^-1^ \| 0.6 ± 0.4 \| 0.3 \| 1.9 \| 0.7 ± 0.4 \| 0.3 \| 1.8 \| 0.7 ± 0.4 \| 0.3 \| 1.8 \| 0.5 ± 0.2 \| 0.2 \| 0.8 \| \| **O_2_ saturation** \| % \| 93.1±25.7 \| 54.9 \| 163.1 \| 78.4±13.7 \| 52.3 \| 115.9 \| 70.4±11.1 \| 52.1 \| 105.3 \| 72.4±8.4 \| 52.7 \| 86.6 \| \| **pH** \| - \| 8 ± 0.2 \| 7.6 \| 8.4 \| 8 ± 0.1 \| 7.7 \| 8.3 \| 7.9 ± 0.1 \| 7.7 \| 8.1 \| 7.8 ± 0.1 \| 7.6 \| 7.9 \| |
|  |
| Table S2. PERMANOVA summary testing the effect of year, month, distance from the sea mouth (Km) and the interaction between year and month on the community composition of Bacteria and Eukaryotes. Abbreviations: df = degrees of freedom, SS= Sum Squares. |
| \| **Community composition Bacteria** \| \| \| \| \| \| **Community composition Eukaryotes** \| \| \| \| \| \| \| --- \| --- \| --- \| --- \| --- \| --- \| --- \| --- \| --- \| --- \| --- \| --- \| \|  \| **df** \| **SS** \| **R^2^** \| **F value** \| **p value** \|  \| **df** \| **SS** \| **R^2^** \| **F value** \| **p value** \| \| **Year** \| 1 \| 0.51 \| 0.02 \| 3.68 \| <0.01 \| **Year** \| 1 \| 0.5 \| 0.01 \| 2.19 \| 0.042 \| \| **Month** \| 1 \| 4.53 \| 0.19 \| 32.53 \| <0.001 \| **Month** \| 1 \| 6.2 \| 0.17 \| 27.18 \| <0.001 \| \| **Km** \| 1 \| 3.62 \| 0.15 \| 25.94 \| <0.001 \| **Km** \| 1 \| 4.83 \| 0.13 \| 21.18 \| <0.001 \| \| **Year x Month** \| 1 \| 0.46 \| 0.02 \| 3.28 \| 0.01 \| **Year x Month** \| 1 \| 0.8 \| 0.02 \| 3.52 \| <0.01 \| \| **Residual** \| 108 \| 15.05 \| 0.62 \|  \|  \| **Residual** \| 106 \| 24.18 \| 0.66 \|  \|  \| \| **Total** \| 112 \| 24.17 \| 1 \|  \|  \| **Total** \| 110 \| 36.51 \| 1 \|  \|  \| |

| Table S3. PERMANOVA summary testing the effect of year, month, distance from the sea mouth (Km) and the interaction between year and month on the ecological function of Bacteria and Eukaryotes. Abbreviations: df = degrees of freedom, SS= Sum Squares. |
| --- |
| \| **Ecological function Bacteria** \| \| \| \| \| \| **Ecological function Eukaryotes** \| \| \| \| \| \| \| --- \| --- \| --- \| --- \| --- \| --- \| --- \| --- \| --- \| --- \| --- \| --- \| \|  \| **df** \| **SS** \| **R^2^** \| **F value** \| **p value** \|  \| **df** \| **SS** \| **R^2^** \| **F value** \| **p value** \| \| **Year** \| 1 \| 0.62 \| 0.02 \| 2.90 \| 0.006 \| **Year** \| 1 \| 0.03 \| 0.00 \| 0.82 \| 0.396 \| \| **Month** \| 1 \| 3.34 \| 0.11 \| 15.68 \| <0.001 \| **Month** \| 1 \| 0.76 \| 0.10 \| 24.39 \| <0.001 \| \| **Km** \| 1 \| 1.87 \| 0.06 \| 8.77 \| <0.001 \| **Km** \| 1 \| 3.58 \| 0.45 \| 115.23 \| <0.001 \| \| **Year x Month** \| 1 \| 0.48 \| 0.02 \| 2.26 \| 0.026 \| **Year x Month** \| 1 \| 0.05 \| 0.01 \| 1.68 \| 0.181 \| \| **Residual** \| 115 \| 24.51 \| 0.80 \|  \|  \| **Residual** \| 113 \| 3.51 \| 0.44 \|  \|  \| \| **Total** \| 119 \| 30.81 \| 1 \|  \|  \| **Total** \| 117 \| 7.93 \| 1 \|  \|  \| |
| 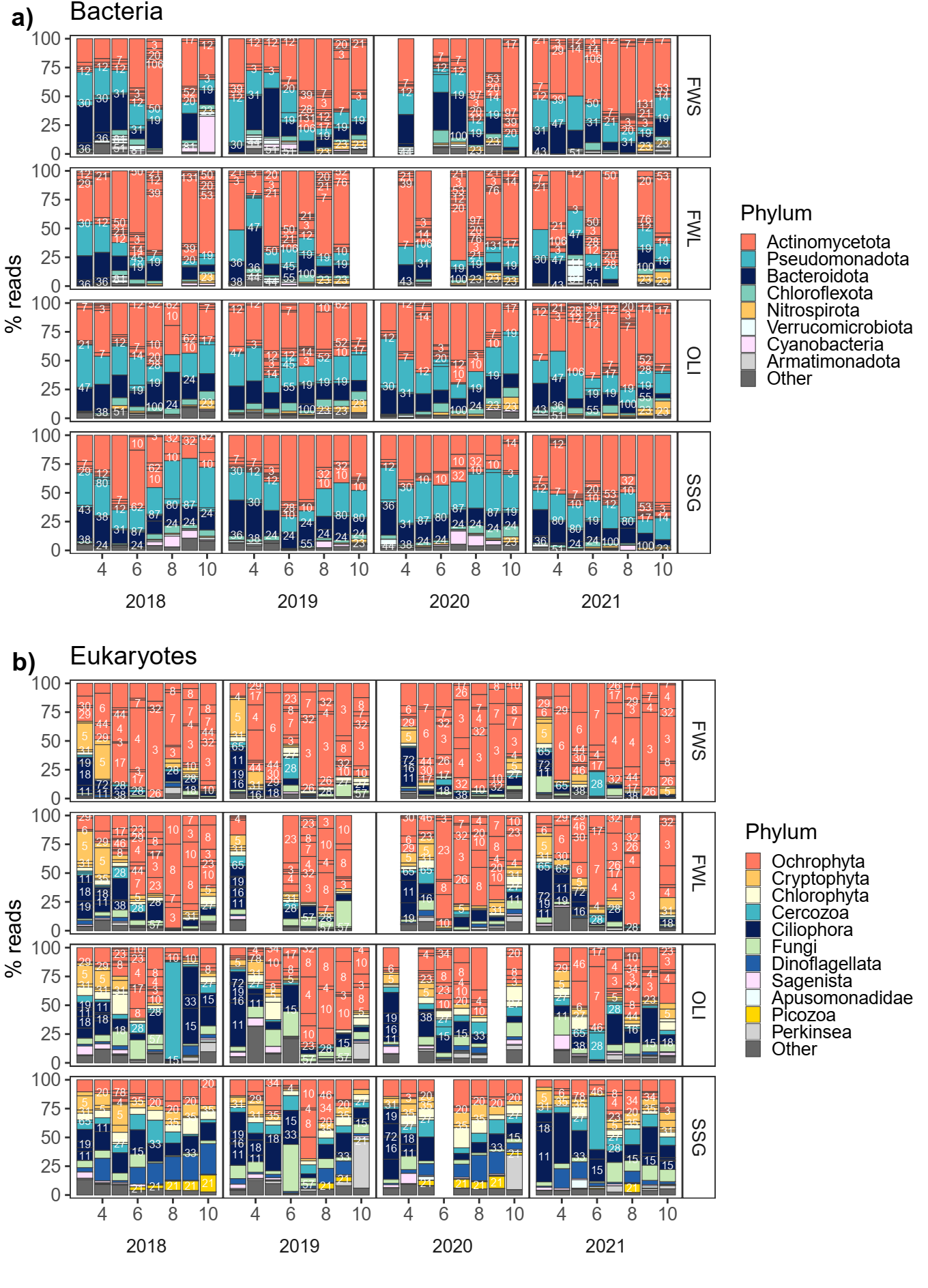 |
| Figure S5. Microbial community structure in the Zeeschelde showing the proportion of reads for the different phyla for a) bacteria and b) eukaryotes. The category others include ASVs with a relative abundance > 1% in more than 5 samples. The numbers on the bars denote the ASV IDs with relative abundance > 1 % in more than 10 samples. Note that those were the taxa included in the NMDs. |
| 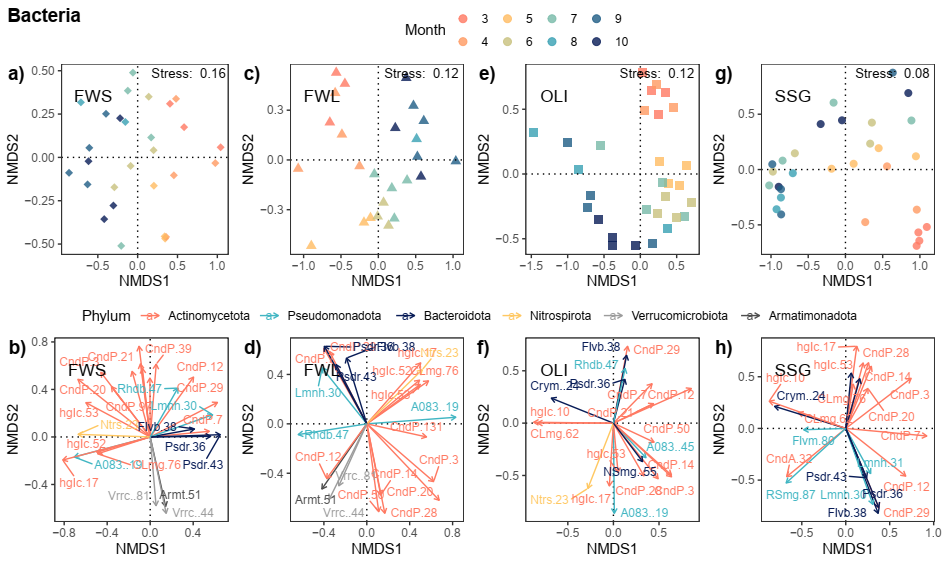 |
| Figure S6. NMDS analysis of Hellinger transformed bacteria FWS (a and b), FWL (c and d), OLI (e and f) and SSG (g and h) from 2018 to 2021. Colors in a, c, e and g indicate the sampling months, in b, d, f and g indicate the phylum. The arrows represent ASVs that significantly (p < 0.05) contribute to explaining the variation in community structure. The full name of the ASVs in b, d, f and g is in Figure 4. |
| 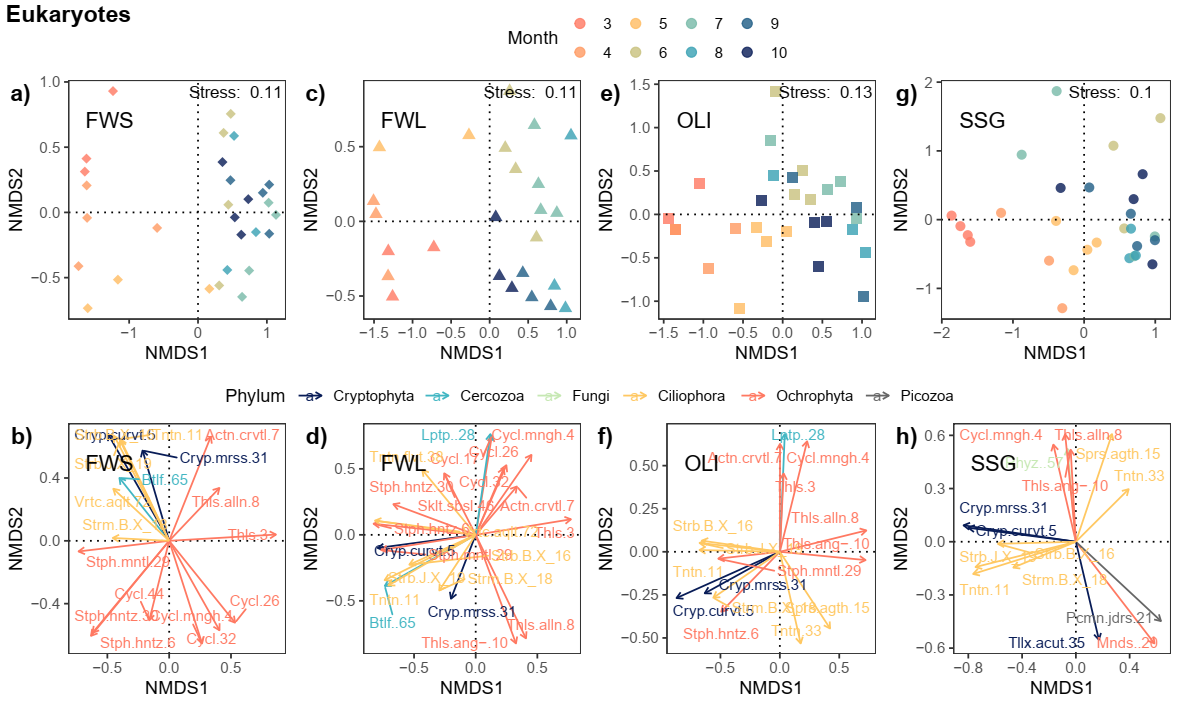 |
| Figure S7. NMDS analysis of Hellinger transformed eukaryotes FWS (a and b), FWL (c and d), OLI (e and f) and SSG (g and h) from 2018 to 2021. Colors in a, c, e and g indicate the sampling months, in b, d, f and g indicate the phylum. The arrows represent ASVs that significantly (p < 0.05) contribute to explaining the variation in community structure. The full name of the ASVs in b, d, f and g is in Figure 5. |
|  |
|  |
| 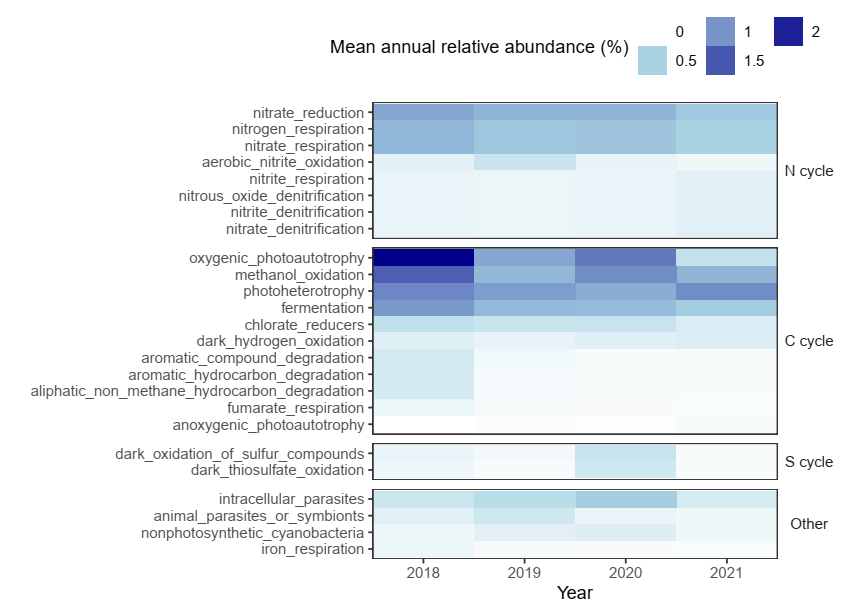 |
| Figure S8. Mean proportion or reads for each ecological function of bacteria using FaProTax in year. |
| 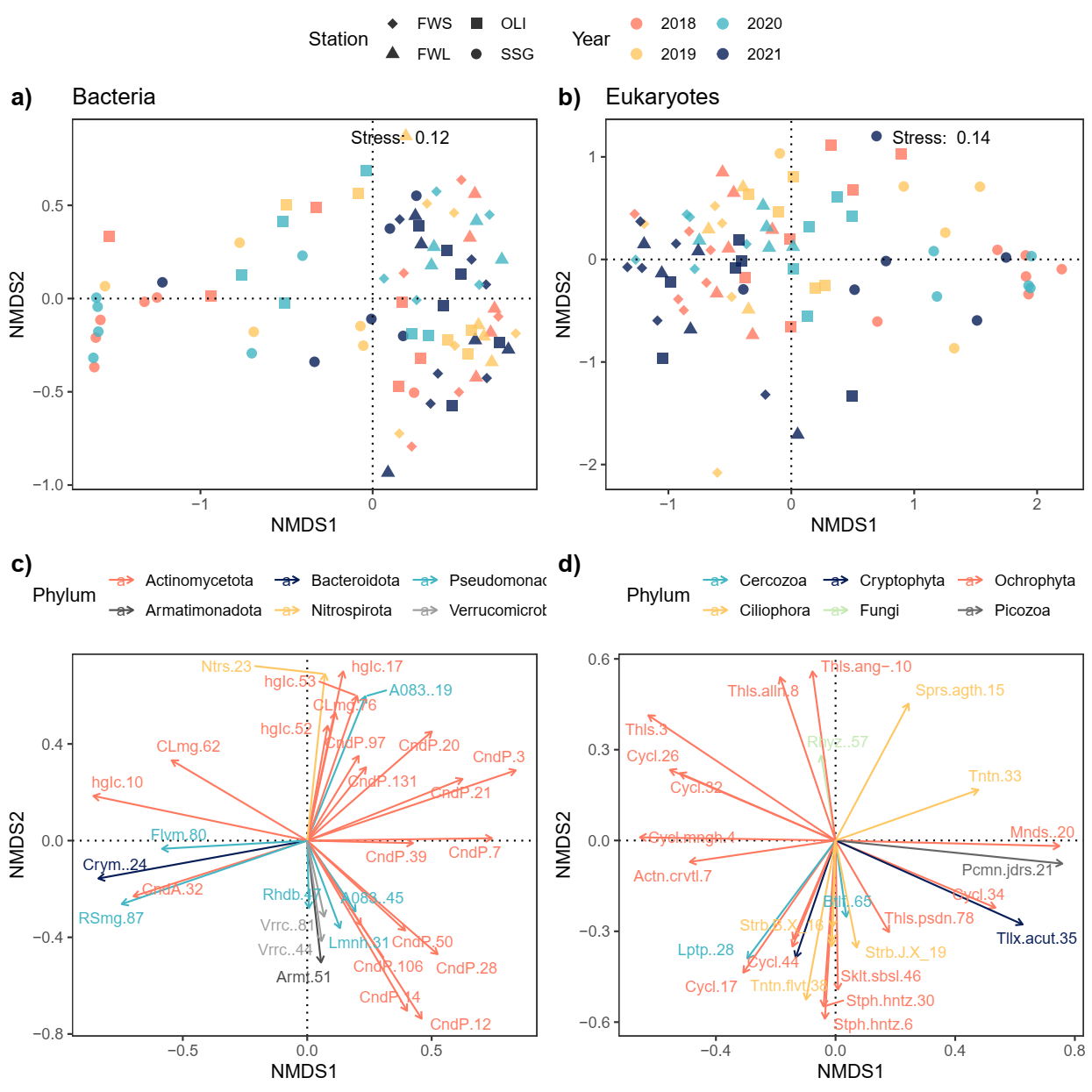 |
| Figure S9. NMDS analysis of Hellinger transformed bacteria (a and c) and eukaryotes (b and d) data from May to October during the study period (2018-2021). The colour of the points (a and b) indicates the year and the shape the station. The arrows represent ASVs that significantly (p < 0.05) contribute to explaining the variation in community structure. |

Figure 5 NMDS analyses of Hellinger transformed eukaryote abundance in the Zeeschelde (2018–2021). a) NMDS plot representing all samples collected throughout the study period. b-e) show individual NMDS plots for each station. Colors indicate the sampling months and symbols the stations. The arrows represent variables that significantly (p < 0.05) contribute to explaining the variation in community structure.

| 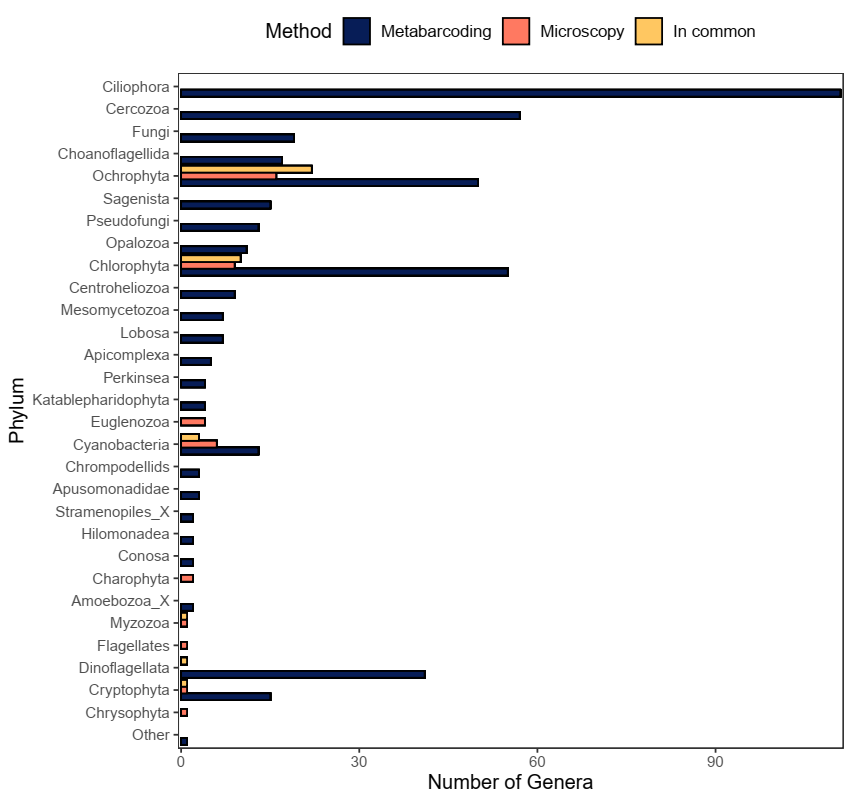 |
| --- |
| Figure S10. Number of eukaryote and cyanobacterial genera identified by metabarcoding (16S and 18S rRNA gene) and microscopy, and number of genera in common to both methods from 2018 to 2021. The category Other combines all the phyla that only presented one genus. |
